# PBMC-derive eNK cells was armed and promoted CAR-like response against tumors through armed with 16Fc21, a novel bispecific fusion protein for NK cells

**DOI:** 10.3389/fimmu.2026.1875554

**Published:** 2026-09-03

**Authors:** Yangyang Li, Yehai Wang, Ximeng Zhang, Yan Fan, Lina Pan, Jing Wu, Weihua Xiao

**Affiliations:** 1 Institute of Advanced Technology, University of Science and Technology of China, Hefei, Anhui, China; 2 Anhui Engineering Research Center of Recombinant Protein Pharmaceutical Biotechnology, Hefei, Anhui, China; 3 Department of Oncology of the First Affiliated Hospital, Division of Life Sciences and Medicine, University of Science and Technology of China, Hefei, Anhui, China; 4 Hefei National Laboratory for Physical Sciences at Microscale, The CAS Key Laboratory of Innate Immunity and Chronic Disease, School of Life Sciences, University of Science and Technology of China, Hefei, Anhui, China; 5 Institute of Immunology, University of Science and Technology of China, Hefei, Anhui, China; 6 Engineering Technology Research Center of Biotechnology Drugs Anhui, University of Science and Technology of China, Hefei, Anhui, China; 7 School of Biological and Food Engineering,Hefei Normal University, Hefei, Anhui, China

**Keywords:** armed-NK, bispecific fusion protein, CAR-NK, natural killer (NK) cell, NK cell immunotherapy, *Pichia (Komagataella) pastoris*

## Abstract

**Introduction:**

Cell-based immunotherapies of PBMC-derive Natural killer (NK) cells have demonstrated substantial potential for the treatment of hematologic malignancies. However, its application is limited due to the complex and inefficient gene modification *in vitro* and the insufficient therapeutic efficacy against solid tumors *in vivo*.

**Methods:**

Herein, a novel bispecific fusion protein 16Fc21(Anti-CD16A-FC-IL21) with capacity of modulate NK cell function is designed and constructed to try to breakthrough these limitations.

**Results:**

In this work, we demonstrated that 16Fc21 has excellent ability to induce the functional activation of PBMC-derived NK cells and improve the therapeutic efficacy of NK cell immunotherapy for solid tumors *in vivo*. Additionally, we developed a novel combination therapy by combining adoptive transfer of NK cell with 16Fc21. We pre-arming 16Fc21 onto the surface of NK cells and successfully developed a non-genetically modified 16Fc21 armedNK (16Fc21 armed-eNK) technology platform.

**Discussion:**

We confirmed that this novel combination therapy can effectively improve the survival and proliferation of NK cells *in vivo*, offering a safer and more effective way to enhance their therapeutic effects *in vivo*. This approach provides a solution to the challenges of the cumbersome *in vitro* gene modification of PBMC-derived NK cells and their insufficient therapeutic *in vivo*.

## Introduction

Cell therapy is one of the emerging tumor treatment methods in recent years (1), showing excellent therapeutic effects in various preclinical and clinical studies, bringing new hope to cancer patients who have been suffering (2–4). In the field of cellular immunotherapy for cancer, the most eye-catching is T cell and NK cell immunotherapy, which are hailed as the twin stars in the field of cancer cell therapy (5). Compared with T cells, NK cells have unique advantages: 1. They can directly kill tumor cells without prior activation; 2. They are not limited by MHC or tumor-specific antigens, allowing them to recognize and kill a variety of tumor cells in a broad spectrum; 3. They have no risk of cytokine storm or neurotoxicity, making them safe and promising as the next generation of broad-spectrum anti-cancer weapons (6, 7).

NK cell adoptive transfer therapy is a treatment that involves collecting NK cells from patients, healthy donors, NK cell lines, or other sources. These cells are then modified and expanded *in vitro* before being infused back into the patient (8–11). Multiple preclinical and early clinical results have shown that NK cell immunotherapy is a safe and effective cancer immunotherapy approach (11–15). It is a rising star treatment method in the field of cancer immunotherapy.

Different sources of NK cells have their own advantages and disadvantages, among which, healthy donor peripheral blood mononuclear cells (PBMC) are highly favored due to their easy acquisition, mature development, short time for *in vitro* expansion and production, and high activity after expansion (16–18). They are one of the important sources of NK cell adoptive transfer therapy. NK cell therapy products based on this source have shown exciting therapeutic effects in clinical and preclinical studies (17, 19, 20). However, despite the fact that PBMC-derived NK cell adoptive transfer therapy has achieved many exciting results so far, its wider clinical application is still limited by several bottlenecks: ① Due to the mature development of PBMC-derived NK cells, they have strong anti-viral activity and high resistance to gene modification based on virus transduction. It is difficult to perform efficient gene modification on NK cells *in vitro* and the production process is complicated and expensive; ② The NK cells infused into the patient’s body exhibit poor infiltration ability toward tumor cells. Moreover, those that reach the tumor site are constrained by the highly immunosuppressive microenvironment within the tumor. This environment restricts the survival and proliferation of NK cells, thereby limiting the duration of their potent anti-tumor activity. As a result, the overall therapeutic effects remain limited. Although many strategies have been developed to address these challenges, they have not yet been effectively resolved. Therefore, urgent measures are needed to explore more solutions or try new approaches to promote the broader clinical application of NK cell immunotherapy (8, 10, 16, 21–24).

CD16A and IL-21R are pivotal activating receptors on natural killer (NK) cells, playing essential roles in their antitumor immune functions. CD16A, a critical mediator of antibody-dependent cellular cytotoxicity (ADCC) (25, 26), initiates robust immune responses when engaged by ligands or antibodies. This activation triggers complex intracellular signaling cascades, resulting in the release of cytolytic molecules such as granzyme and perforin, as well as pro-inflammatory cytokines and chemokines, which collectively enhance NK cell cytotoxicity and antitumor efficacy. Furthermore, this process upregulates the expression of key receptors associated with NK cell activation, survival, and proliferation, including CD69, NKG2D, 4-1BB, and IL-21 (27–30). These changes enhance NK cells’ sensitivity to corresponding ligands or therapeutic antibodies, providing a basis for developing advanced NK cell-based immunotherapies. IL-21 plays a multifaceted role in modulating NK cell function. Through the JAK/STAT and PI3K/MAPK pathways (31), IL-21 enhances the effector activity of NK cells in both human and murine models. Although IL-21 inhibits NK cell proliferation in murine models, it simultaneously promotes their activation, maturation, and the production of cytolytic molecules (e.g., perforin and granzyme), ultimately boosting cytotoxic activity (32). In human NK cells, IL-21 demonstrates similar enhancements, effectively promoting NK cell activation, survival, and proliferation *in vitro* (33). Moreover, IL-21 synergizes with other cytokines, such as IL-15, to further enhance NK cell cytotoxicity, stimulate perforin and granzyme production, and induce IFN-γ secretion (33, 34). Notably, IL-21 has been shown to augment ADCC-mediated NK cell cytotoxicity. For example, Roda et al. reported that IL-21 significantly enhanced the ability of NK cells to eliminate HER2-positive breast cancer cells (35). Recent studies further revealed that CAR-NK cells engineered to express autocrine IL-21 exhibited superior survival, proliferation, and therapeutic efficacy against glioblastoma *in vivo* (36). These findings collectively underscore the critical role of IL-21 in promoting NK cell survival, proliferation, cytokine secretion, and cytotoxicity, highlighting the potential of leveraging IL-21 in combination with CD16A to develop next-generation NK cell-based immunotherapies.

In this work, we successfully developed a novel bispecific fusion protein, 16Fc21, designed to simultaneously target CD16A and IL-21R on NK cells. 16Fc21 effectively induced NK cell activation, and demonstrated robust antitumor efficacy *in vivo*. Traditional bispecific antibody therapies often face challenges due to the low abundance and suboptimal functional state of NK cells in patients, limiting their therapeutic potential (8, 37, 38). Therefore, we developed a novel strategy involving adoptive transfer of NK cells pre-arming with 16Fc21. By leveraging the CD16A-targeting antibody fragment within 16Fc21, we successfully pre-arming NK cells *in vitro*, creating non-genetically modified 16Fc21 armed-eNK cells. This innovative therapeutic approach significantly enhanced NK cell survival and proliferation *in vivo*, improving antitumor efficacy while maintaining safety.

These findings highlight the potential of 16Fc21 and 16Fc21 armed-eNK cells as transformative therapeutic modalities to overcome critical bottlenecks in PBMC-derived NK cell-based immunotherapy. Our study provides a solid foundation for future development of novel immunotherapy strategies base PBMC-derived NK with enhanced clinical efficacy and safety profiles.

## Results

### Design, construction and expression of 16Fc21

**Figure 1:**
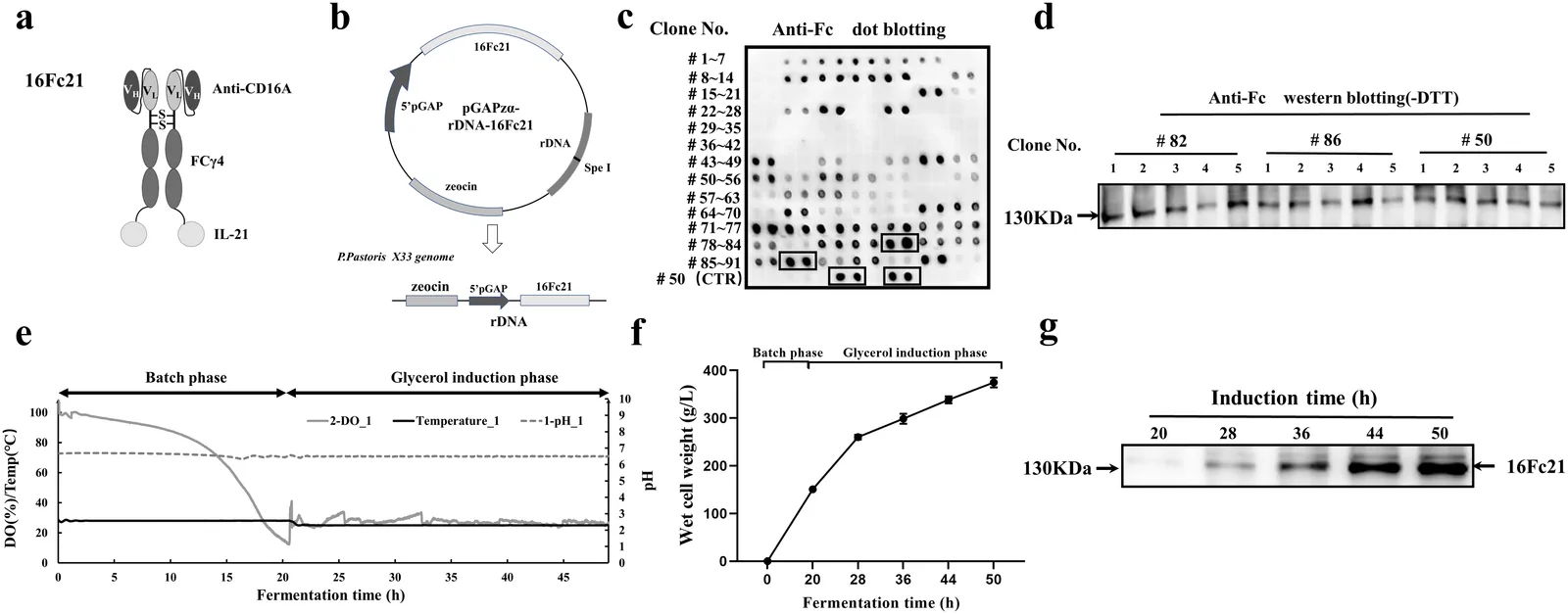
Construction and expression of 16Fc21. **(a)** Schematic of the 16Fc21 molecular structure, which is a bispecific recombinant protein formed as a homodimer through the interchain disulfide bonds of an IgG4 Fc; this protein fuses a single-chain variable fragment derived from the human CD16A antibody with human cytokine IL-21. **(b)** The 16Fc21 coding sequence was cloned into the pGAPzα-rDNA plasmid under the control of the GAP promoter and integrated into the *P. pastoris* X33 genome via homologous recombination. **(c)** Dot blot analysis with an anti-human Fc HRP-conjugated antibody identified positive colonies expressing 16Fc21. **(d)** High-expression clones were further evaluated by Western blot under non-reducing conditions (-DTT), clone #82–5 exhibited the highest expression level and was selected for subsequent studies. **(e–g)**. A typical fermentation process was selected for display. **(e)** Monitoring of 16Fc21 expression *Pichia pastoris* fermentation process, DO: dissolved oxygen; Temp: temperature; **(f)** Changes in wet cell weight of the strain during fermentation; **(g)** Western blot results showing the accumulation of 16Fc21 protein in the supernatant over time.

In this study, we designed a bispecific recombinant fusion protein (Anti-CD16A-FC-IL21), named 16Fc21 (**Figure 1a**), that specifically targets CD16A and IL-21R on NK cells. 16Fc21 consists of a single-chain Anti-CD16A antibody, human cytokine IL-21, and IgG4 Fc, with the IgG4 Fc mediating the formation of a homodimer through inter-chain disulfide bonds. The Anti-CD16A arm of 16Fc21 binds to CD16A on NK cells, leading to NK cell activation, while the IL-21 moiety binds to IL-21R, further modulating NK cell functional status to enhance its antitumor activity. Additionally, the use of IgG4 Fc fragment without physiological functions in 16Fc21 has been shown to increase the *in vivo* half-life of molecules containing this fragment in murine models (39).

We expressed 16Fc21 using a homologous recombination approach in *K. phaffii*, transforming the linearized 16Fc21 expression vector into the wild-type *K. phaffii* strain X33 (**Figure 1b**). Positive transformants were selected on Zeocin-containing YPD plates, and expression levels were initially assessed by dot blot analysis. Among 91 positive transformants, clones 82 and 86 exhibited strong signals, with clone 82 showing higher expression levels compared to the previously selected clone 50 (**Figure 1c**). Further analysis by Western blot confirmed that clone 82–5 demonstrated superior expression and stability. Although the expressed protein in this clone exhibited some aggregation, this could potentially be mitigated through optimization of downstream processing, suggesting that 82–5 is the most suitable clone for further production and research (**Figure 1d**).

### Optimization of fermentation parameters for 16Fc21 at shake-flask scale

To optimize the production of 16Fc21 at a 10 L fermentation scale, we first refined the culture conditions for *K. phaffii* expressing 16Fc21. We investigated the effects of pH and temperature on 16Fc21 production by culturing 16Fc21-expressing strains in BMGY medium with varying pH (6.0, 6.5, 7.0). After a 60-hour cultivation period, we observed that 16Fc21 accumulated in the supernatant under all three pH conditions, with the highest expression occurring at pH 6.5 (**Supplementary Figure 1A**). Based on this result, we selected pH 6.5 for subsequent fermentations.

Next, we evaluated the impact of temperature on 16Fc21 expression by cultivating the yeast at 20 °C, 25 °C, and 30 °C in BMGY with pH 6.5. The highest protein accumulation occurred at 20 °C, but this condition also resulted in the highest proportion of aggregated proteins. At 25 °C, the yield was similar to 20 °C, but the aggregation was lower, making it the optimal temperature for large-scale fermentation (**Supplementary Figure 1B**).

To further enhance 16Fc21 expression, we explored the effect of glycine supplementation, as 16Fc21 contains a GS linker that may require additional glycine for optimal expression. Adding 3 mM glycine to the culture medium significantly increased 16Fc21 accumulation in the supernatant (**Supplementary Figure 1C**). Additionally, the inclusion of 2 g/L sodium citrate further boosted 16Fc21 production, with no significant enhancement when both glycine and citrate were added together (**Supplementary Figures 1D, E**).

### Pilot-scale fermentation, purification, and characterization of 16Fc21

Building on the optimized shake-flask conditions, we established a 10 L fermentation process based on Invitrogen’s *K. phaffii* fermentation guide. The fermentation process consisted of two phases: a glycerol batch phase (20 hours) followed by a glycerol-fed induction phase (30 hours). Key parameters, including temperature, pH, and dissolved oxygen (DO), were monitored in real time (**Figure 1e**). Throughout the fermentation, the yeast culture exhibited continuous growth, reaching a final cell wet weight (WCW) of 377 g/L after 50 hours (**Figures 1e, f**). During the glycerol-fed induction phase, 16Fc21 accumulated steadily in the supernatant, reaching a plateau after 24 hours (**Figure 1g**).

**Figure 2:**
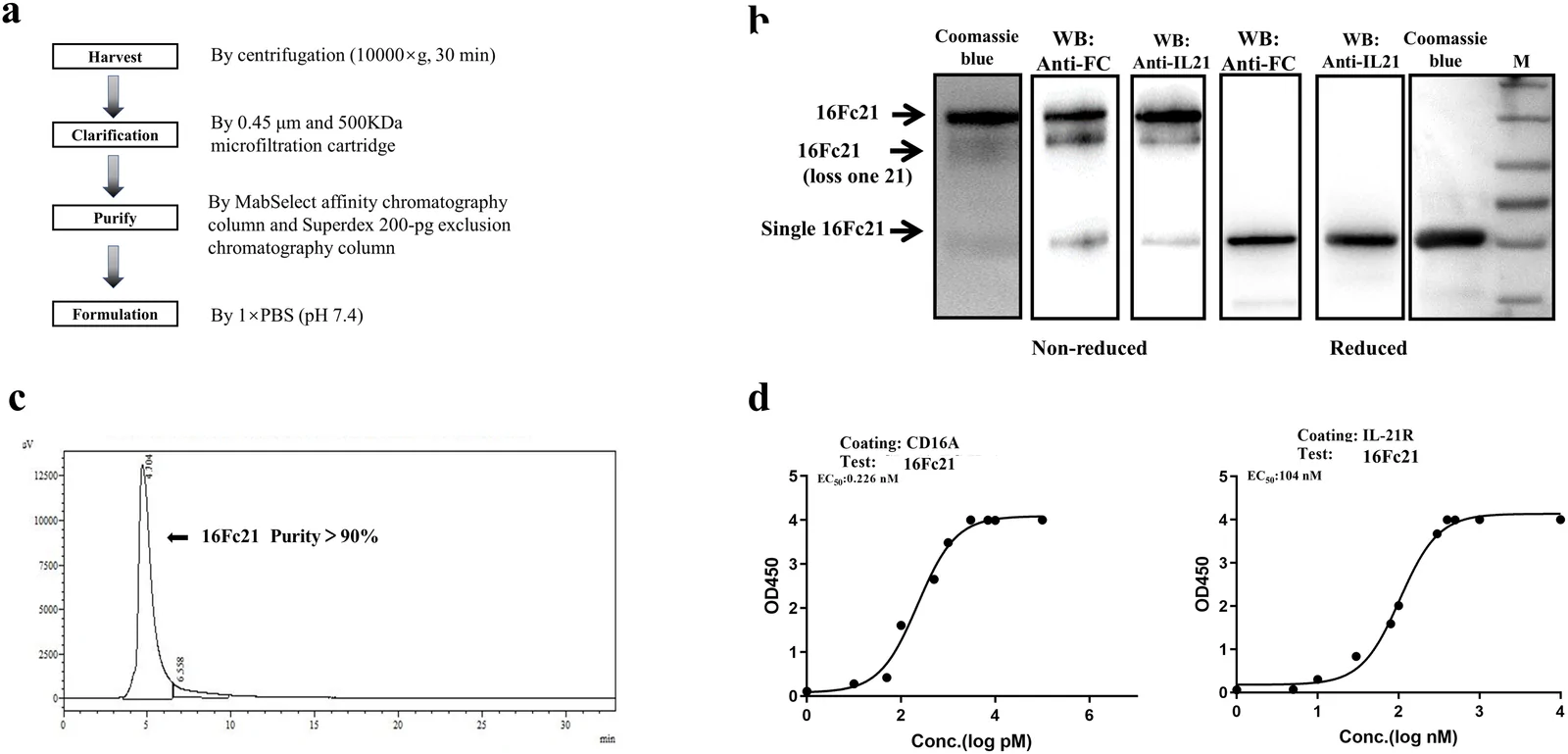
Purification, and characterization of 16Fc21. **(a)** Purification process flow chart of 16Fc21; **(b)** SDS-PAGE and Western blotting were used to identify the physicochemical property of 16Fc21 protein; **(c)** The purity of 16Fc21 protein was determined with SEC-HPLC; d Affinity of 16Fc21 for CD16A receptor (left) and IL-21 receptor (right).

Following fermentation, the supernatant was collected after centrifugation and filtration, and 16Fc21 was purified using a HiTrap MabSelect affinity column, followed by size-exclusion chromatography on a Superdex S200 column (**Figure 2a**). The final 16Fc21 product, with a purity >90%, was analyzed by SDS-PAGE, Western blot and HPLC (**Figures 2b, c**). We identified two smaller degradation products beneath the main 16Fc21 band, likely resulting from IL-21 domain truncation or disulfide bond cleavage (**Figure 2b**).

To evaluate the *in vitro* activity of 16Fc21, we performed ELISA to measure its binding affinity to human CD16A and IL-21 receptors. 16Fc21 demonstrated strong binding to both targets, with EC50 values of 0.226 nM for CD16A and 104 nM for IL-21R (**Figure 2d**). These results confirm that 16Fc21 retains its expected biological activity and can effectively bind to both of its target receptors.

#### 16Fc21 effectively activates PBMC-derived NK cells

**Figure 3:**
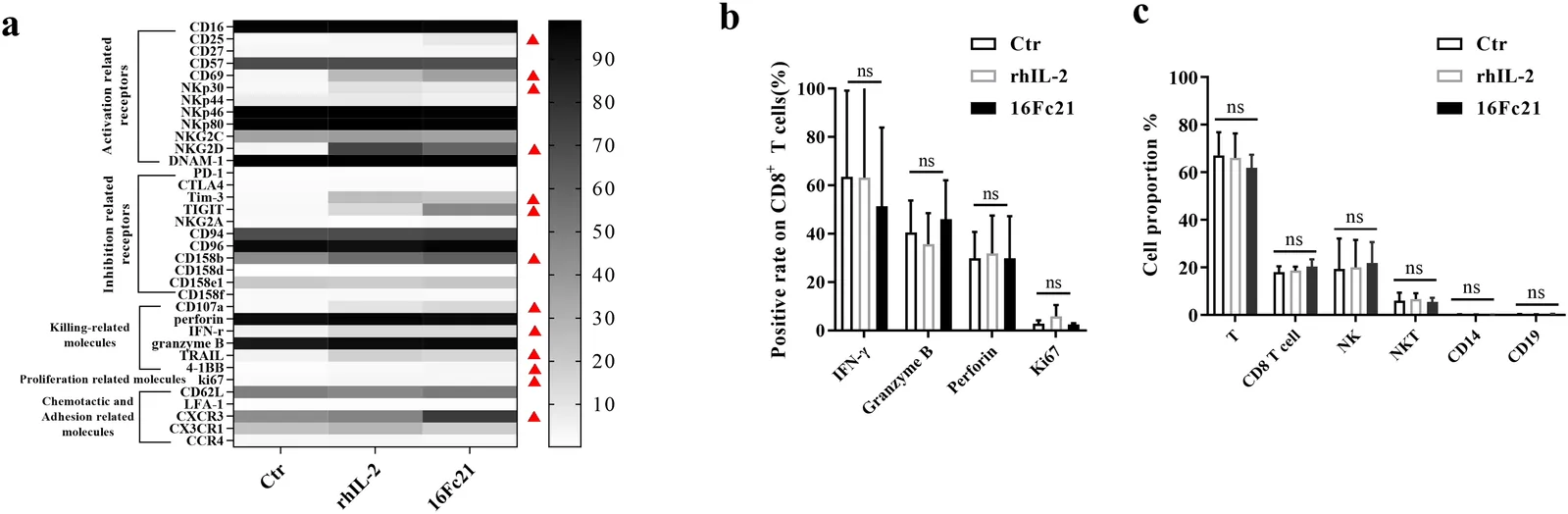
16Fc21 can effectively activate PBMC-derived NK cells. PBMCs from healthy donors (n=5) were co-cultured with PBS, 50 IU/mL rhIL-2, and 20 nM 16Fc21 for 24 hours. Flow cytometry was used to detect the expression levels of the molecules in PBMC-derived NK cells and CD8^+^ T cells. **(a)** The effect of 16Fc21 on the expression of different functional molecules on the surface of PBMC-derived NK cells; **(b)** The effect of 16Fc21 on the expression of different functional molecules on the surface of PBMC-derived CD8^+^ T cells. **(c)** The effect of 16Fc21 on the proportion of cell subpopulations in PBMCs. Significant differences were determined by comparing each group with the PBS group, ns p>0.05.

To assess the functional activity of 16Fc21, we co-incubated it with fresh PBMCs isolated from healthy donors. After 24 hours, we analyzed NK cell activation using flow cytometry. 16Fc21 significantly increased the expression of key activation markers (NKG2D, CD69, NKp30, CD25) and cytotoxic molecules (CD107a, IFN-γ, 4-1BB, TRAIL), inhibitory molecules (Tim-3, TIGIT, CD158b), indicating that 16Fc21 effectively activates NK cells (**Figure 3a**). Notably, 16Fc21 also upregulated proliferation marker Ki67, suggesting a potential for promoting NK cell survival and proliferation. Moreover, 16Fc21 treatment did not significantly affect CD8+ T cell markers or cell subset distribution (**Figures 1b, c**). These findings demonstrate that 16Fc21 can effectively activate PBMC-derived NK cells and enhance their functionality.

#### 16Fc21 can pre-arming onto the surface of PBMC-derived expanded NK, and the 16Fc21 armed-eNK have enhanced functional activity and anti-tumor ability

**Figure 4:**
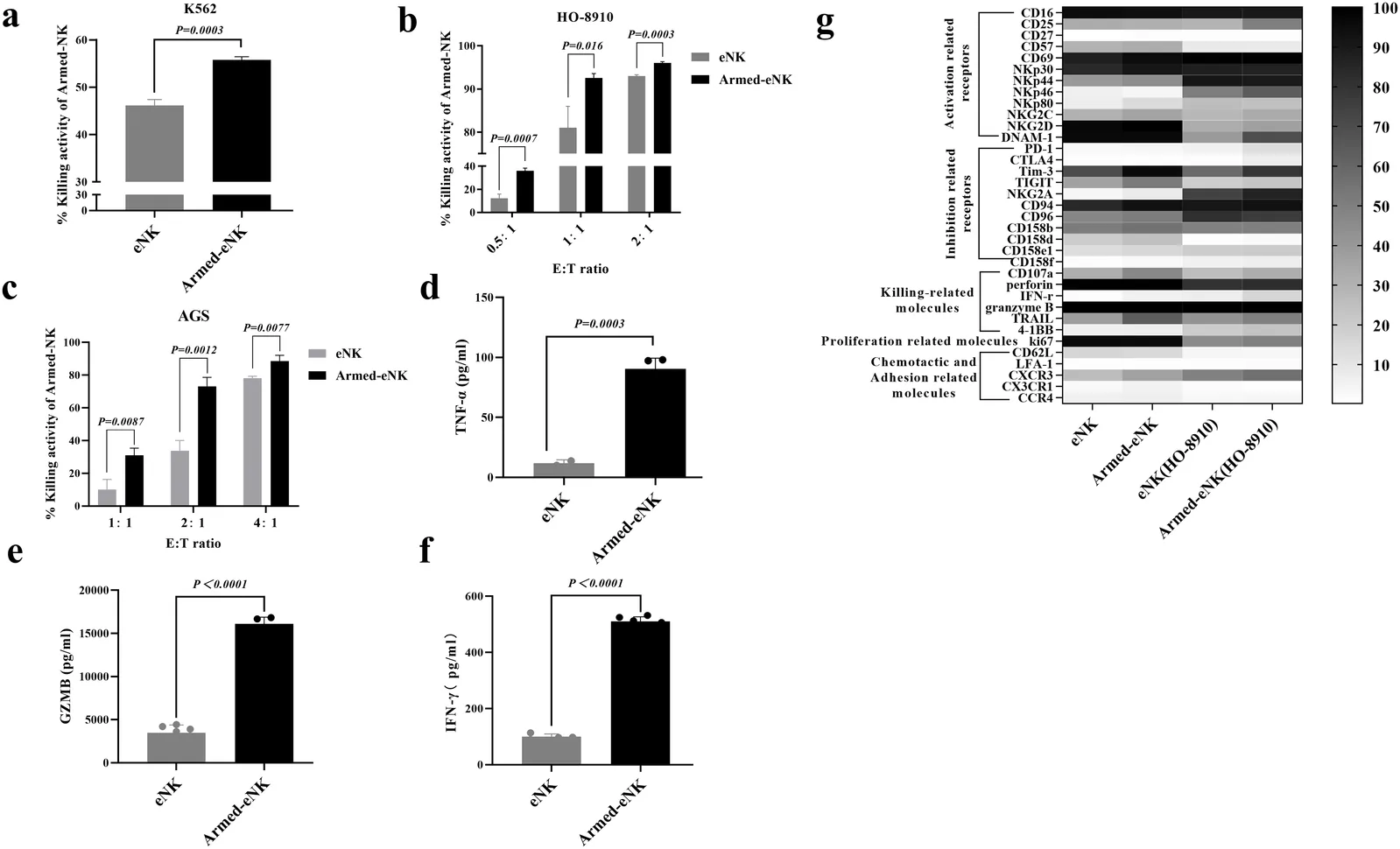
16Fc21 armed-eNK exhibit strong anti-tumor activity. 16Fc21 was pre-loaded onto the surface of expanded NK cells *in vitro*, conferring CAR-NK-like effects. The functionality of the 16Fc21 armed-eNK cells was assessed by measuring their cytotoxicity and the expression of functional molecules. **(a)** 16Fc21 armed-eNK demonstrate stronger cytotoxicity against K562 (E:T=1:1). **(b)** Armed-eNK demonstrate stronger cytotoxicity against HO-8910 tumor cells at different E:T ratio. **(c)** Armed-eNK demonstrate stronger cytotoxicity against AGS tumor cells at different E:T ratio. **(d–f)** Monitor the changes in the content of functional cytokines in the supernatant of the co-culture of Armed-eNK and HO-8910 cells. **(g)** Monitoring of functional molecule expression in the Armed-eNK cells with or without tumor cells (HO-8910) (n = 3).

To further explore the practical application value and potential of 16Fc21, we investigated the feasibility and efficacy of 16Fc21 pre-arming PBMC-derived expanded NK cells. We first explored and established an *in vitro* incubation system for 16Fc21 armed-eNK cells. When the pre-arming incubation system was at 4 °C, the 16Fc21 concentration was 100 μg/mL, and the NK cell concentration was not greater than 5 × 10^8 cells/mL, 16Fc21 could be effectively pre-arming onto NK cells with the highest 16Fc21 signal detectable on the NK cell surface after 1 hour of incubation under gentle mixing (**Supplementary Figure 2**). So, does the 16Fc21 armed-eNK cell have better anti-tumor activity? To investigate this, we used K562 and HO-8910 as target cells to test the anti-tumor activity of 16Fc21 armed-eNK cells. We found that 16Fc21 armed-eNK cells more effectively killed K562 , HO-8910 and AGS cells, suggesting that 16Fc21 armed-eNK cells exhibit superior anti-tumor activity (**Figures 4a–c**). To further explore the functional activities changes of 16Fc21 armed-eNK cells, we cultured/co-cultured 16Fc21 armed-eNK cells without/with human ovarian cancer cells HO-8910 under a low-efficiency target ratio (1:1), simulating the physiological conditions of low-efficiency target ratios in tumor environments. We observed that 16Fc21 armed-eNK exhibited significantly enhanced functional activity regardless of the presence of tumor cells. Multiple related functional molecules were markedly upregulated, including various activating (CD25; NKp44; NKp46; NKp80), inhibitory (PD-1; CTLA4; NKG2A; CD96), cytotoxic (IFN-γ; 4-1BB), chemotactic and adhesion molecules (CXCR3). When co-cultured with tumor cells, the expression of these NK cell-associated functional molecules was further elevated, suggesting that 16Fc21 pre-arming NK cells possess substantially enhanced functional activity (**Figure 4f**).

Furthermore, we analyzed changes in cytokine levels in the culture supernatant under co-culture conditions with tumor cells. It was found that the concentrations of TNF-α, granzyme B (GZMB), and IFN-γ were significantly increased in the co-culture supernatant of the 16Fc21-pre-armed NK cell group, indicating that 16Fc21 armed-eNK possesses markedly enhanced anti-tumor activity (**Figures 4c–e**). These findings are consistent with the results obtained from the cytotoxicity assays described earlier.

In summary, these results demonstrate that 16Fc21 armed-eNK cells exhibit significantly enhanced functional activity and anti-tumor capacity.

#### 16Fc21 armed-eNK cells exhibit outstanding anti-tumor capabilities *in vivo*

**Figure 5:**
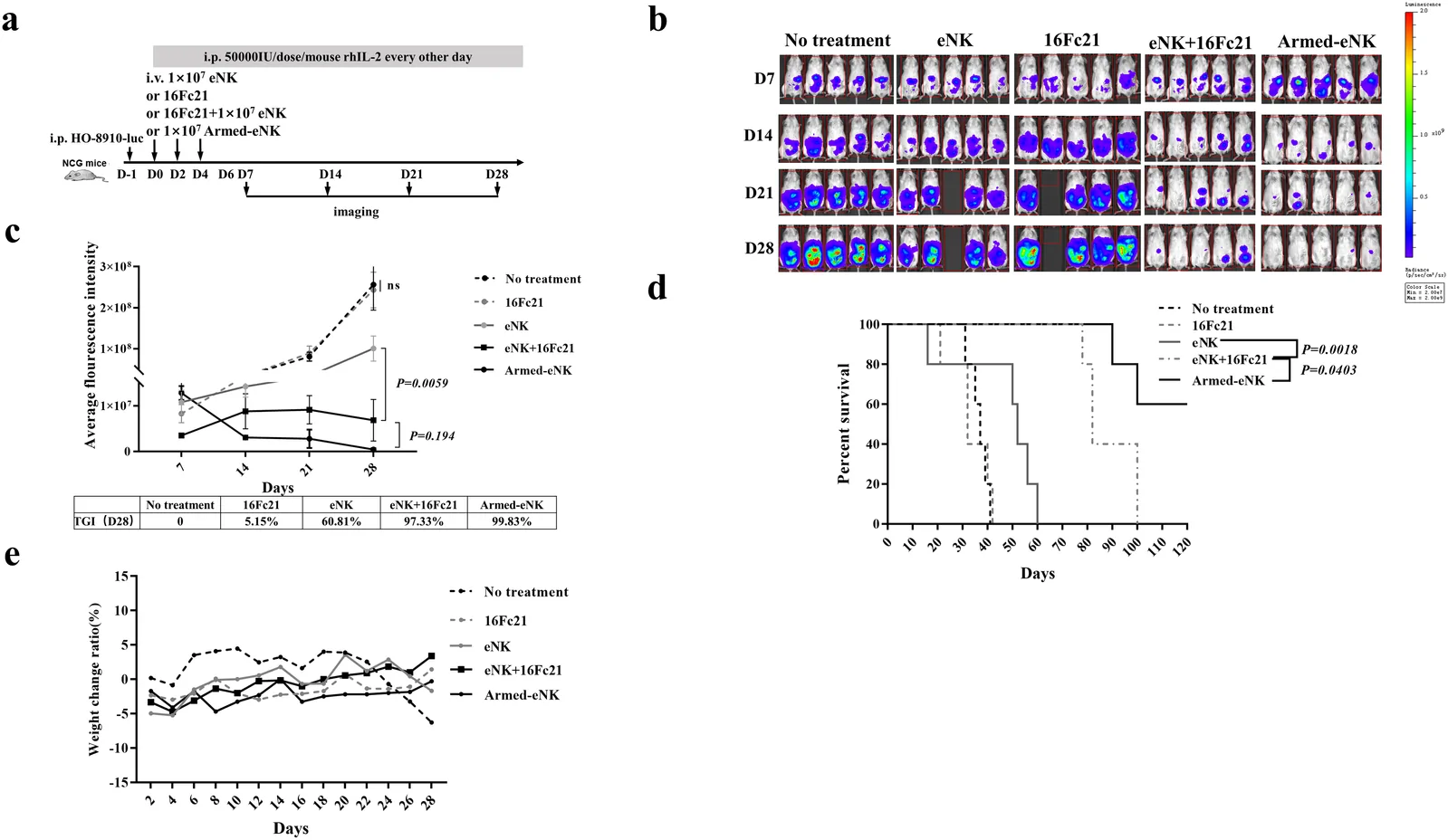
16Fc21 armed-eNK cells exhibit strong growth inhibition on HO-8910-LUC Abdominal cavity tumor mouse model. **(a)** Experimental design for the xenograft model of human ovarian cancer HO-8910-Luc. **(b)** Imaging results showing tumor development at different time points. **(c)** Monitoring of tumor average fluorescence intensity at different time points. **(d)** Survival analysis of mice in each group. **(e)** Changes in body weight of mice during the experiment. (n=5). ns, not significant.

To further evaluate the anti-tumor activity of 16Fc21 armed-eNK cells *in vivo*, we conducted pharmacodynamic experiments using human ovarian cancer NSG mouse models. We infused either normal NK cells or 16Fc21 armed-eNK cells to treat the mice and verify whether the 16Fc21 armed-eNK cells have better *in vivo* anti-tumor activity (**Figure 5a**). We found that both normal NK cells and 16Fc21 armed-eNK cells effectively inhibited ovarian cancer progression in the mice, with 16Fc21 armed-eNK cells showing better anti-tumor activity, leading to more significant inhibition of ovarian cancer development (**Figures 5b, c**). Furthermore, the capacity of 16Fc21 armed-eNK to control tumor progression was the most potent among all experimental groups. Under these experimental conditions, 16Fc21 armed-eNK not only effectively suppressed tumor growth but also achieved near-complete tumor eradication. In this group, the tumor burden in all mice was significantly lower than that on day 7 (D7), and tumors in three mice were nearly eliminated (**Figures 5b, c**). Correspondingly, these three mice survived for more than 120 days (**Figure 5d**). These results indicate that 16Fc21 armed-eNK exhibits significantly enhanced *in vivo* anti-tumor activity. Additionally, mice in the combination therapy group receiving eNK along with 16Fc21 demonstrated tumor control and clearance effects similar to those observed in the 16Fc21 armed-eNK group, although the overall therapeutic efficacy was inferior to that of 16Fc21 armed-eNK (**Figures 5b, c**). This suggests that the superior therapeutic effect of 16Fc21 armed-eNK is not merely attributable to the simple combination of its components, but rather reflects the function of a novel protein–cell complex, analogous to CAR-NK, which possesses unique therapeutic activity and practical application value (**Figures 5b, c**).

Furthermore, intravenous injection of 16Fc21 alone did not significantly affect tumor development. After treatment with 16Fc21 armed-eNK cells, the survival period of the mice was significantly prolonged compared to the NK cell treatment group, with three mice surviving beyond 120 days (**Figure 5d**). The mice did not show significant weight loss compared to before treatment (**Figure 5e**), suggesting that 16Fc21 armed-eNK cells have favorable safety.

**Figure 6:**
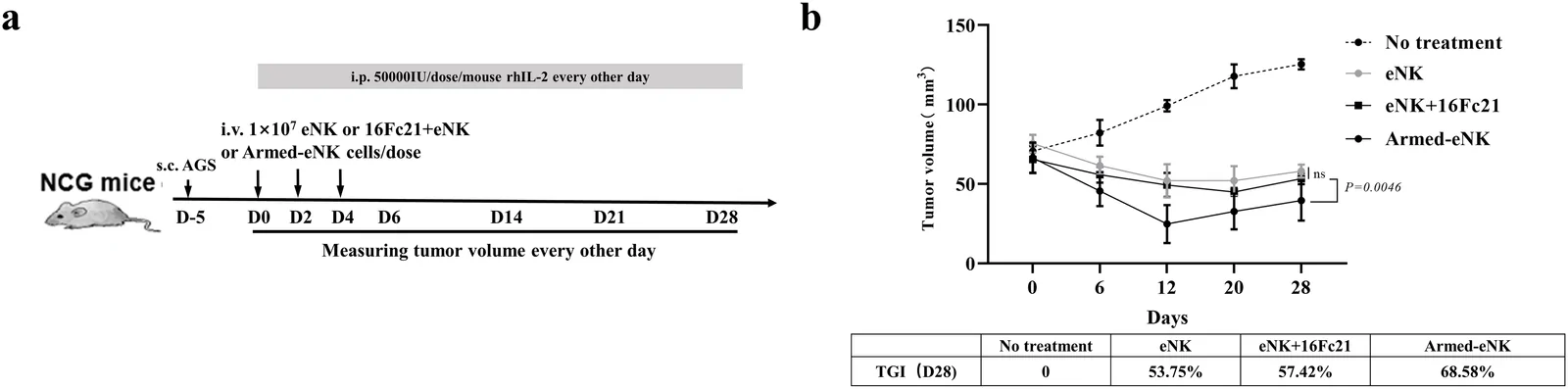
16Fc21 armed-eNK cells exhibit strong growth inhibition on AGS subcutaneous tumor mouse model. **(a)** Experimental design for the subcutaneous human gastric cancer AGS model. **(b)** Measurement of tumor volume in mice at different time points (n=5).

Additionally, we further tested the *in vivo* anti-tumor activity of 16Fc21 armed-eNK cells using a subcutaneous gastric cancer mouse model (**Figure 6**). We found that NK cell infusion effectively controlled gastric cancer development in the subcutaneous tumor-bearing mice, and armed-eNK cells demonstrated superior therapeutic effects compared to the NK + 16Fc21 group (**Figure 6b**), indicating that the superior anti-tumor activity of 16Fc21 armed-eNK cells is not merely a combination effect.

In conclusion, these results suggest that 16Fc21 armed-eNK cells have superior *in vivo* anti-tumor efficacy, and the application of 16Fc21 armed-eNK cells is more valuable than the direct combination of 16Fc21 with NK cells.

#### 16Fc21 armed-eNK show enhanced proliferation in xenograft ovarian tumor-bearing NSG mice

**Figure 7:**
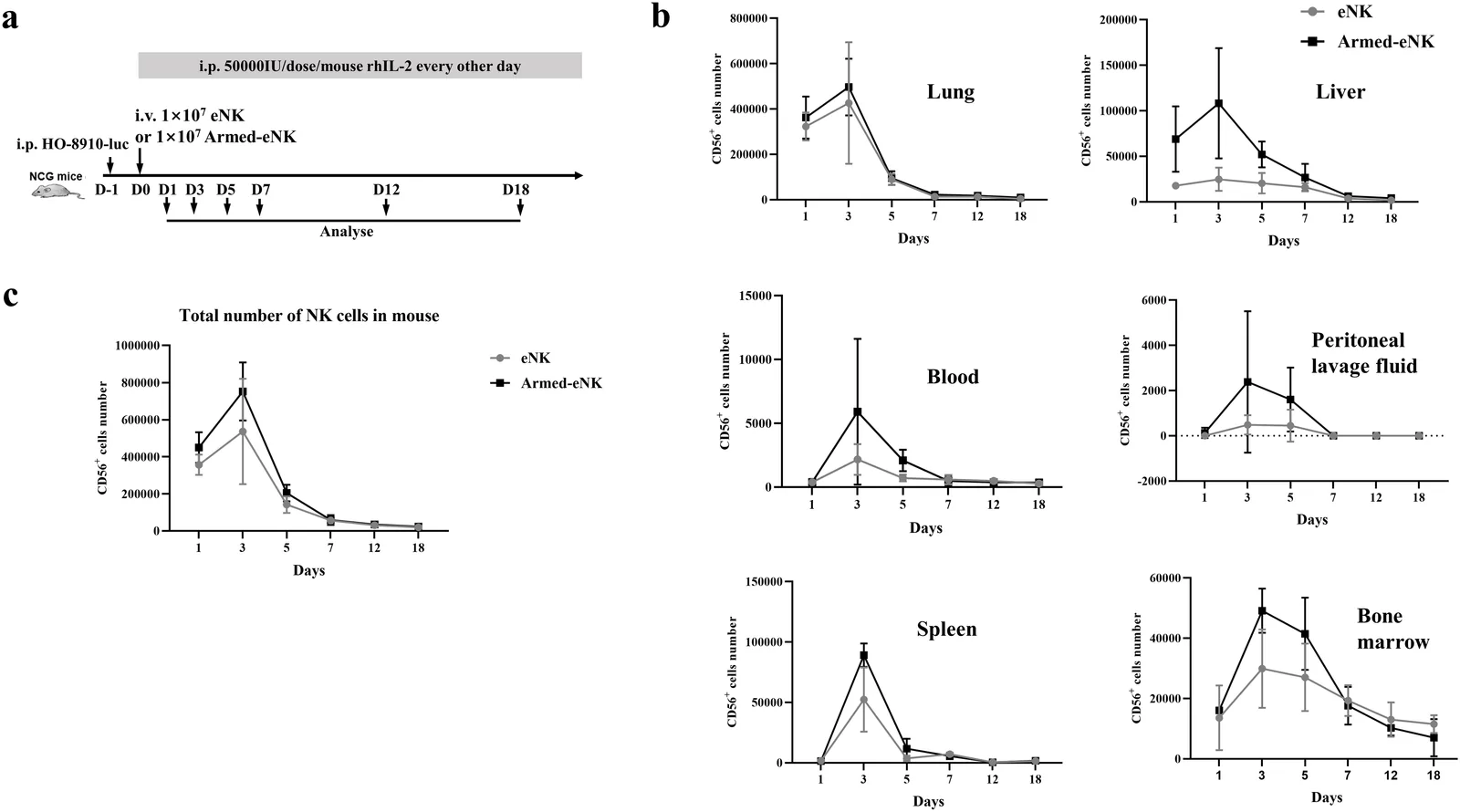
16Fc21 armed-eNK cells induce eNK cell proliferation in *in vivo*. **(a)** Experimental design for *in vivo* survival and proliferation activity assessment. **(b)** Detection of cell infiltration in various organs. **(c)** Summary of cell infiltration results in various organs. (n=6).

Proliferation, survival, and activity of NK cells in the tumor environment are well-established as essential factors contributing to their anti-tumor efficacy *in vivo*. To further explore the deeper reasons for the excellent *in vivo* efficacy of 16Fc21 armed-eNK, we re-infused 16Fc21 armed-eNK cells into NCG mice bearing HO-8910 tumors and then isolated immune cells from different organs of the mice to assess NK cell survival and proliferation via flow cytometry (**Figure 7a**). *In vivo* proliferation kinetics of adoptively transferred cells in NCG mice showed that NK cells primarily localized to lung and liver tissues, followed by spleen, bone marrow, blood, and peritoneum (**Figure 7b**). The number of human CD45^+^CD56^+^ cells peaked at Day 3, remaining stable for approximately 10–14 days. Notably, in all six samples, we found that the abundance of adoptively transferred NKs peaked at the same time in both groups. However, a higher number of human CD45^+^CD56^+^ cells were observed in the 16Fc21 armed-eNK group, both in various organs and in the total NK cell count within the mice (**Figures 7b, c**), suggesting that 16Fc21 armed-eNK cells have improved survival and proliferation *in vivo*. This likely contributes to the superior therapeutic effects of 16Fc21 16Fc21 armed-eNK cells.

#### 16Fc21 armed-eNK have good safety *in vivo*

**Figure 8:**
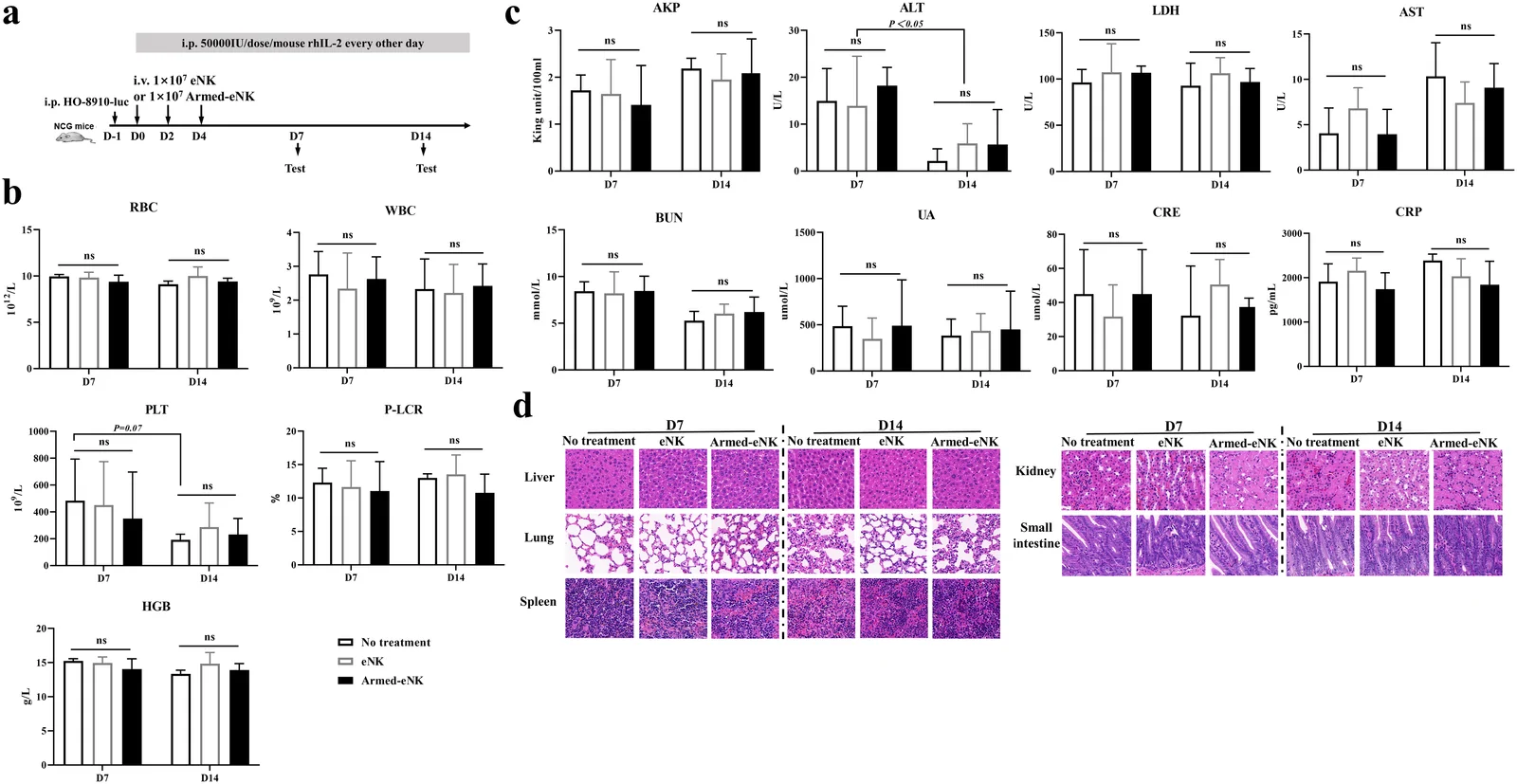
Safety evaluation of 16Fc21 armed-eNK cells in mice. **(a)** Experimental protocol for *in vivo* safety evaluation. **(b)** Hematological analysis results. **(c)** Results of biochemical blood tests. **(d)** Immunohistochemical analysis of organ damage. (n=5). ns, not significant.

Given that 16Fc21 armed-eNK cells show enhanced anti-tumor activity, does this improved therapeutic effect introduce additional safety risks? To evaluate whether the safety of 16Fc21 armed-eNK cell infusion therapy is compromised compared to unarmed NK cells, we conducted a safety assessment using the previously described pharmacodynamic model (**Figure 8a**). The main safety concern with cell infusion therapy is graft-versus-host disease (GVHD), which can cause organ dysfunction or damage. Therefore, we conducted biochemical blood tests and immunohistochemical assays to assess organ damage in different tissues of the mice, evaluating the health of mice receiving 16Fc21 armed-eNK cell infusion. We found no significant health changes in the mice treated with either 16Fc21 armed-eNK or unarmed NK cells. The key biochemical indicators and organ damage were comparable to those of normal mice (**Figures 8b, c**). Furthermore, GVHD reactions typically result in increased white blood cell counts and anemia in mice (40). To explore this, we analyzed the blood of these mice and found no significant decrease in white blood cells (WBC), red blood cells (RBC), platelets (PLT), or hemoglobin (HGB) in the mice treated with 16Fc21 armed-eNK cells compared to unarmed NK cells (**Figure 8c**). In addition, we did not observe any significant tissue damage in the mice treated with reinfusion of 16Fc21 armed-eNK. The morphology of all organs remained intact and normal (**Figure 8d**; **Supplementary Figure 3**). These results indicate that while 16Fc21 armed-eNK cells enhance anti-tumor activity, they do not introduce additional safety risks, suggesting that 16Fc21 16Fc21 armed-eNK cells have an acceptable safety profile.

## Discussion

In this study, we designed and developed an NK cell bispecific recombinant fusion protein, 16Fc21 (Anti-CD16A-FC-IL21), targeting the CD16A and IL-21R receptor molecules on NK cells. We chose the cheap and stable *K. phaffii* expression system to successfully express this bispecific NK cell recombinant fusion protein. Subsequently, through a series of shake flask and 10 L fermentation tank optimization experiments, we established a stable 10 L fermentation tank-based protein production process that can be used for the stable and efficient production of high-purity recombinant protein. *In vitro* experiments showed that the recombinant protein produced under this process could stably bind to its corresponding receptors and effectively activate NK cells derived from human peripheral blood mononuclear cells (PBMCs), upregulating the expression of activation, cytotoxicity, and proliferation-related molecules on the NK cell surface. This indicates that 16Fc21 can enhance NK cell anti-tumor activity and promote NK cell proliferation and survival, proving the feasibility of using *K. phaffii* to express complex therapeutic proteins.

Further, we explored the feasibility and efficacy of 16Fc21-enhanced adoptive NK cell transfer therapy. By pre-arming NK cells with 16Fc21 *in vitro*, we obtained a novel NK cell therapy product—16Fc21 armed-eNK cells. Through both *in vitro* and *in vivo* experiments, we demonstrated that this new 16Fc21 armed-eNK cell therapy product not only had better anti-tumor activity but also exhibited good *in vivo* safety, indicating its great clinical application potential.

NK cell activity is determined by the integration of signals from both inhibitory and activating receptors on their surface (38, 41). When NK cells are in a strongly inhibitory tumor microenvironment, higher levels of inhibitory signals suppress NK cell activity, hinder their anti-tumor function, and lead to immune exhaustion (42). Currently, there are two main approaches to reversing the exhausted state of immune cells in the tumor microenvironment: one is to block inhibitory signals, such as the PD-1/PD-L1 immune checkpoint, and the other is to provide activating signals to NK cells to counteract the immunosuppressive effects caused by inhibitory signals (8, 43). CD16A is a major activating receptor on NK cells and a key target for developing drugs that enhance NK cell function. Many CD16A-based drugs, including monoclonal antibodies and bispecific/multispecific antibodies, have been developed to enhance NK cell anti-tumor activity. In this study, we selected an Anti-CD16A antibody to target and enhance NK cell activity, and additionally targeted the IL21 receptor molecule on NK cells to further enhance NK cell activation, proliferation, and survival, thereby improving NK cell anti-tumor activity.

To verify whether 16Fc21 has the expected biological activity, we treated PBMCs from healthy donors with 16Fc21. We found that 16Fc21 effectively activated NK cells from PBMCs, leading to upregulation of activation molecules (CD69, CD25, NKG2D, NKp30), cytotoxic molecules (4-1BB, CD107a, TRAIL), and proliferation-related molecules (Ki67) on the NK cell surface, suggesting that 16Fc21 effectively activates NK cells. Moreover, 16Fc21 significantly increased the expression of NK-related inhibitory molecules Tim-3 and TIGIT, which suggests that 16Fc21 may have a stronger activation effect on NK cells. The activity of NK cells is determined by the overall effect of both activation and inhibitory molecules on their surface. To prevent NK cells from killing healthy host cells, NK cells have evolved to express inhibitory molecules like MHC molecules that suppress the killing of healthy cells (44, 45). Thus, the increase in Tim-3 and TIGIT expression in the 16Fc21 treatment group might indicate a stronger intended activation effect.

CAR-NK is an area of high interest in NK cell immunotherapy (10, 24). However, current CAR-NK cell development, based on peripheral blood-derived NK cells, is limited by the highly mature developmental state of peripheral blood NK cells, making genetic modification challenging (46). This is one of the technological bottlenecks restricting the further application of peripheral blood-derived NK cells. Furthermore, the CAR-NK production process is complicated and costly, and the viral transfection methods commonly used for modifying NK cells can potentially introduce genomic alterations, posing safety risks (47–49). To address this, we further explored and developed 16Fc21 pre-arming (armed) eNK cells. We established a simple *in vitro* pre-arming system for NK cells derived from peripheral blood after expansion, which only takes two hours to modify NK cells without complex genetic modification or lengthy production processes, greatly reducing production costs.

One of the challenges limiting NK cell immunotherapy for some malignant tumors is the insufficient efficacy of this treatment, which is a major hurdle for its wider application (10, 22, 50). NK cells infused into patients are often limited by the highly immunosuppressive tumor microenvironment, leading to poor survival and proliferation, and preventing them from sustaining anti-tumor activity as expected (21, 42, 51). Enhancing NK cells’ ability to resist the immunosuppressive tumor microenvironment is therefore key to improving the efficacy of NK cell immunotherapy. In this study, we observed that NK cells pre-arming with 16Fc21 exhibited significantly enhanced *ex vivo* and *in vivo* activity as well as superior anti-tumor efficacy, leading to more efficient tumor cell killing. In co-culture experiments with tumor cells *ex vivo*, multiple functional molecules associated with NK cell activity were markedly upregulated, including various activating, inhibitory, cytotoxic, chemotactic, and adhesion-related molecules. Furthermore, *in vivo* studies demonstrated that 16Fc21 armed-eNK cells also displayed potent anti-tumor activity, effectively controlling and eliminating tumors in mice. Importantly, this therapeutic superiority was not merely attributable to the simple combination of 16Fc21 and NK cells, but rather reflected the formation of a novel protein–cell complex resembling a CAR-NK entity, which possesses unique therapeutic potential and practical applicability. Additionally, pharmacokinetic studies *in vivo* revealed enhanced proliferative capacity of 16Fc21 armed-eNK cells, suggesting that the improved anti-tumor activity of 16Fc21 armed-eNK stems from a comprehensive enhancement of functional properties. This includes directly augmented cytotoxic ability, indirectly elevated secretion of anti-tumor cytokines, and enhanced proliferative activity, collectively contributing to the overall functional improvement of NK cells. Notably, despite this broad functional enhancement, 16Fc21 armed-eNK did not introduce additional safety risks in mice.

Both *in vitro* and *in vivo* experiments show that 16Fc21 armed-eNK cells have superior anti-tumor activity and safety, suggesting their great application value and potential for further clinical research. However, the current experiments were conducted using immune-deficient NSG mice, which lack a complete immune system. Although we observed encouraging results in treating ovarian cancer mice, it is not yet clear whether similar outcomes will be achieved in real patients. Additionally, the potential for additional safety risks remains uncertain and will require further comprehensive studies.

In summary, in this study, we designed and developed the bispecific NK cell fusion protein 16Fc21 and demonstrated its excellent biological activity through a series of experiments. We explored the application of 16Fc21, the development of an *in vitro* pre-arming system for peripheral blood-derived NK cells, providing support for further clinical applications of 16Fc21 and 16Fc21 armed-eNK cells.

## Materials and methods

### Strains, plasmids, and antibodies

The *Komagataella phaffii* (*K. phaffii*) strain X33 and *Escherichia coli* strain TOP10 were purchased from Invitrogen (Carlsbad, CA, USA). The expression vector pGAPzα-rDNA was constructed and stored in our laboratory. HRP-conjugated anti-His antibody (A00612) was purchased from Genscript.

Fluorescently conjugated antibodies used for phenotypic analysis of NK cells included anti-LFA-1 (363404), anti-CXCR3 (353720), anti-PD-1 (329906), anti-CD107a (328612), anti-NKP44 (325116), anti-CD158e1 (312706), anti-CD158d (347006), anti-CD27 (356412), anti-CCR4 (359412), anti-DNAM-1 (338316), anti-CD16 (302040), anti-Granzyme B (515406), anti-CD62L (304806), anti-CD69 (310910), anti-NKP80 (346706), anti-NKP30 (325210), anti-CD158f (341304), anti-CD158b (312612), anti-Tim-3 (345012), anti-CD94 (305504), anti-TIGIT (372706), anti-TRAIL (308206), anti-CD57 (322306), anti-CX3CR1 (341610), anti-NKG2D (320808), anti-Perforin (353310), anti-Ki67 (350504), anti-IFN-γ (506518), anti-CD94 (305506), anti-NKp46 (331916), anti-CTLA4 (369614), anti-CD96 (338416), anti-41BB (309818), and anti-CD25 (356108), all purchased from BioLegend. Anti-CD159a/NKG2A (FAB1059P) and anti-NKG2C (FAB138G) were purchased from R&D Systems. Fluorescently conjugated antibodies for immune cell identification in expanded NK or PBMC populations included anti-human Lineage Cocktail (348803), anti-CD56 (362550), anti-CD3 (300430), anti-CD33 (366620), anti-HLA-DR (307606), anti-CD14 (301836), anti-CD19 (302242), anti-CD11b (301322), anti-CD25 (356108), and anti-FOXP3 (320108), all purchased from BioLegend, San Diego, CA, USA.

### Mice and cell lines

Female NCG (NOD/ShiLtJGpt-Prkdc^em26Cd52^Il2rg^em26Cd22^/Gpt) mice were purchased from Jiangsu Gempharmatech. Mice were maintained in accordance with institutional guidelines approved by the Animal Research Committee of the University of Science and Technology of China and housed in an ultraclean barrier facility. The expanded NK cells derived from PBMC used in this study were all provided by Shanghai NK CellTech Co., Ltd. The K562 (RRID: CVCL_0004) cell line was obtained from the Cell Bank of the Chinese Academy of Sciences (Shanghai, China). The Ho8910-Luc cell line was constructed and cryopreserved in our laboratory. All cell lines were cultured in modified RPMI medium (HyClone, SH30809.01) supplemented with 10% FBS (Biological Industries, Beit HaEmek, Israel), 100 U/mL penicillin, and 100 µg/mL streptomycin (Sangon Biotech, Shanghai, China) at 37 °C in a 5% CO_2_ incubator. Cell line identity was confirmed by STR profiling (Genewiz, Suzhou, China).

### Molecular design and construction of expression vectors

The scFvCD16A-Fc sequence was obtained from our lab, while the IL-21 domain was sourced from NCBI. The 16Fc21 fusion gene was constructed by linking scFvCD16A-Fc and IL-21 via a GS linker. Codon optimization was performed, and restriction sites for XhoI and NotI were added at both ends of the fusion gene. The synthesized sequence was inserted into the Zeocin-resistant vector pGAPzα-rDNA, generating the expression vector pGAPzα-rDNA-16Fc21.

### Screening for expression

The pGAPzα-rDNA-16Fc21 expression vector was linearized with SpeI and electroporated into *K. phaffii* X33 cells. Transformed cells were plated on YPD agar plates containing 300 µg/mL Zeocin (Sigma). Positive clones were screened for protein expression by dot blot and Western blotting. The clone with the highest expression was selected for pilot-scale fermentation.

### Fermentation and purification

A two-step fermentation was performed using a 14-L NBS BioFlo fermenter (Eppendorf, Hamburg, Germany) following Invitrogen’s Pichia Fermentation Process Guidelines and our previous work (52). In the first stage, 400 mL of *K. phaffii* seed culture was added to 6 L of BMGY medium containing 4% (w/v) glycerol to initiate fermentation. The temperature was maintained at 28 °C, and pH was controlled at 6.5. During the glycerol induction phase, 50% (w/v) glycerol feed containing 12 mL/L PTM1 solution was added to induce recombinant protein synthesis. Oxygen was controlled at 25% air saturation, stirring speed was set to 1000 rpm, and the induction phase lasted no longer than 30 hours to prevent protein degradation. After fermentation, the supernatant was collected by centrifugation and filtered through 0.45-µm and 500-kDa microfiltration cartridges. Protein was captured by MabSelect affinity chromatography, followed by size-exclusion chromatography using HiLoad 26/600 Superdex 200-pg exclusion columns on an AKTA Pure25 system (GE Healthcare Life Sciences). Purity was assessed by SDS-PAGE, Western Blot and HPLC, and endotoxin levels were measured using the Tachypiens Amebocyte Lysate (TAL) assay (Bioendo, Fujian, China), yielding endotoxin levels < 0.01 EU/μg protein. The purified protein was filtered through 0.22-µm filters, aliquoted, and stored at −80 °C.

### Measurement of binding ability by ELISA

Human CD16A (R&D, 0425-FC) or IL-21 Receptor (Genscript, Z03382) (1 μg/mL) was coated on 96-well Corning plates and incubated overnight at 4 °C. After washing with PBST, the plate was blocked with 5% BSA in PBS for 1 hour. Following incubation with varying concentrations of 16Fc21 for 2 hours, the plate was washed and incubated with HRP-conjugated anti-Fc detection antibody for 1 hour. After further washing, the developer solution was added, and absorbance was measured at OD450 using a plate reader. Data were analyzed using GraphPad Prism.

### PBMC isolation and *in vitro* culture

PBMCs were isolated from healthy donor blood by Ficoll-Hypaque density gradient centrifugation with informed consent (Anhui Blood Center). PBMCs were cultured in RPMI medium supplemented with 10% FBS and treated with 50 IU/mL rhIL-2, 20 nM 16Fc21 for 24 hours. Flow cytometry analysis was performed as previously described (53).

### NK cell receptor expression analysis

NK cells were harvested and washed twice with PBS. Cells were resuspended in PBS containing 10% mouse serum for 15 minutes at 4 °C to block Fc receptors, followed by incubation with appropriate antibodies for 30 minutes at 4 °C. For intracellular cytokine detection (perforin, Granzyme B, and IFN-γ), cells were pretreated with 2.5 ng/mL monensin (Sigma) for 4 hours. All antibodies were purchased from BioLegend. Dead cells were excluded using DAPI, and receptor expression levels were analyzed by flow cytometry on a Beckman Coulter Cytoflex.

### 16Fc21 pre-arming NK cells for *in vitro* non-gene modification

Expanded NK cells derived from PBMCs were washed twice with pre-chilled 1× PBS (500 × g, 5 min) and resuspended in physiological saline containing 1% human serum albumin. The cell concentration was adjusted to 1 × 10^7^ cells/mL, and 20 µL of the cell suspension was taken for counting. 16Fc21 protein was added to the suspension to achieve a final concentration of 100 µg/mL, followed by incubation with low-speed shaking at 4 °C for 1 hour. After incubation, the cells were analyzed for 16Fc21 loading using FITC-conjugated anti-Fc antibody by flow cytometry.

#### Flow-based killing assay

K562 cells (1 × 10^6^) were collected and incubated with 5 μM CFSE (BioLegend, San Diego, CA, USA, Cat. No. 423801) at 37 °C for 15 minutes in the dark. After washing three times, the cells were resuspended in 1 ml RPMI 1640 medium with 10% FBS. Then, 100 μl was added to the reaction system, so that the final number of K562 cells in the reaction system was 0.1×10^6^ (54). The 16Fc21 armed-eNK cells were co-incubated with K562 cells at E:T=1:1 for 24 hours at 37 °C. Propidium iodide (BioLegend, Cat. No. 421301) was added for the last 5 minutes at room temperature to label dead cells. Specific killing of K562 cells by NK cells was assessed by flow cytometry (Beckman Coulter).

#### RTCA-based killing assay

This assay was performed using the Real-Time Cell Analysis (RTCA) system (ACEA Biosciences, San Diego, CA, USA) according to the manufacturer’s instructions. HO-8910 cells were washed twice, resuspended in RPMI 1640 containing 10% FBS, and seeded at 2 × 10^4^ cells/well in a 100 µL volume. Cells were cultured in a constant temperature incubator at 37 °C for 13 hours. 16Fc21 armed-eNK cells were washed twice and resuspended in RPMI 1640 medium with 10% FBS, and co-incubated with HO-8910 cells at different E:T ratio for 24 hours at 37 °C. The killing effects were monitored in real-time using the RTCA system.

### Mouse tumor models

#### Intraperitoneal ovarian cancer mouse model

*In vivo* experiments were performed according to the guidelines of the Animal Research Committee of the University of Science and Technology of China. Female NCG mice (8–10 weeks old) were intraperitoneally injected with 8 × 10^5^ HO-8910-Luc cells per mouse on the designated day. Mice were then randomly divided into several experimental groups (five mice per group) for subsequent treatments. NK cells were injected via the tail vein, and combination therapy drugs were administered by intraperitoneal injection, including IL-2, which is a commonly used administration route for ovarian cancer murine models (53, 55).

Tumor burden was monitored by bioluminescence imaging. Mice were injected intraperitoneally with D-luciferin (150 µg/g) in PBS, anesthetized by inhalation of 2% isoflurane (flow rate 2.5–3 L/min), and imaged using the IVIS Spectrum Imaging System (PerkinElmer, Boston, MA, USA). Tumor progression was monitored on days 7, 14, 21, and 28.

#### Subcutaneous gastric cancer model

Female NCG mice (8–10 weeks old) were subcutaneously injected with 6 × 10^6^ AGS cells per mouse on the indicated days. Mice were then randomly divided into experimental groups (five mice per group). NK cells were injected through the tail vein, and combination therapy drugs were administered intraperitoneally, as described for the ovarian cancer model. Tumor growth was measured every two days to monitor its development.

### Analysis of NK cell distribution in NCG mice

Female NCG mice bearing ovarian cancer were injected with 1 × 10^7^ NK cells in 250 µL of PBS. Additionally, mice received intraperitoneal injections of rhIL-2 (50,000 IU/mouse) every other day. At the time of sacrifice(carrying out euthanasia through cervical dislocation method), tissues including spleen, liver, and lungs were collected. An automatic tissue grinder (Miltenyi Biotec, Bergisch Gladbach, Germany) was used as per the manufacturer’s instructions.

For spleen tissue, 5 mL of PBS was added and crushed with the m_spleen_01 program. Lungs were dissected into 1–2 mm³ pieces and digested with RPMI 1640 containing 1 IU/mL DNase I (Sigma, D5025) and 10 µg/mL collagenase IV (Sigma, St. Louis, MO, USA, C5138) for 1.5 hours at 37 °C, followed by grinding with the m_lung_01 program. Liver tissue was processed similarly in RPMI 1640 with DNase I and collagenase IV and crushed with the 37 °C_m_LDK_01 program. Bone marrow was flushed using an injector, and peripheral blood was collected. Blood cells were lysed using red blood cell lysis buffer (Solarbio, Beijing, China, R1010) for 5 minutes at room temperature. The peritoneal fluid was harvested by irrigation with PBS.

Single-cell suspensions from these tissues were washed twice and resuspended in PBS containing 10% mouse serum at 4 °C for 15 minutes. The cells were incubated with the following antibodies for 30 minutes at 4 °C: anti-human FITC-CD3 (BioLegend, Cat. No. 300406), anti-human PE/Cy7-CD56 (Cat. No. 318318), anti-human APC/Cy7-CD45 (Cat. No. 368516), and anti-mouse PerCP/Cy5.5-CD45 (Cat. No. 103132). All antibodies were purchased from BioLegend.

### Histopathological evaluation and blood analysis

Following euthanasia, the organs of the test mouse were collected and fixed in 4% paraformaldehyde for 24 hours, followed by rinsing with double-distilled water overnight. The tissues were then sequentially dehydrated in graded ethanol solutions: 50% for 15 minutes, 70% for 15 minutes, 80% for 30 minutes, 95% for 30 minutes, and 100% for 15 minutes, with the entire process repeated once. After ethanol dehydration, the tissues were immersed in xylene for 1 hour. The dehydrated tissues were then embedded in paraffin to form blocks. The paraffin-embedded tissues were sectioned as required and baked in a slide dryer for 1 hour. The sections were subsequently immersed in xylene three times, each for 15 minutes, followed by immersion in absolute ethanol for 5 minutes, repeated once. The sections were then placed in 85% ethanol for 5 minutes and subsequently in 75% ethanol for 5 minutes, after which they were rinsed with distilled water. Antigen retrieval was performed by placing the sections in citrate antigen retrieval buffer and heating in a microwave oven. After cooling to room temperature, the sections were rinsed three times with 1× PBS. Endogenous peroxidase activity was blocked by incubation in hydrogen peroxide at room temperature for 25 minutes, followed by three washes with 1× PBS, each for 5 minutes. The sections were then blocked with 3% BSA for 30 minutes. After gentle blotting to remove the blocking solution, the sections were incubated with primary antibodies in a humidified chamber at 4 °C overnight. The sections were washed three times with 1× PBS, each for 5 minutes, and then incubated with secondary antibodies at room temperature for 50 minutes. Following three additional washes with 1× PBS (each for 5 minutes), freshly prepared DAB chromogen solution was applied. Upon completion of chromogenic staining, the reaction was terminated by rinsing with double-distilled water. The sections were then sequentially dehydrated in 75% ethanol, 85% ethanol, and absolute ethanol for 5 minutes each, with the process repeated once. The sections were cleared in xylene, air-dried, mounted with neutral balsam, and coverslipped. Microscopic examination and image acquisition were subsequently performedImages were obtained using a ZEISS Axioskop2 plus advanced positive microscope.

For blood analysis, mice blood was collected into EDTA-coated tubes and analyzed using an automatic blood analyzer (SYSMEX, Tokyo, Japan, XT-1800i) according to the manufacturer’s instructions. Serum samples were collected and analyzed using an automatic biochemical analyzer as per the manufacturer’s guidelines.

### Statistical analysis

GraphPad Prism 7.0 software was used to perform statistical analysis in this study. The statistical significance was determined by one-way analysis of variance (ANOVA). The survival curve was analyzed using the log-rank (Mantel–Cox) test. Data are presented as the means ± SDs (standard deviation). ns: not significant; *: p < 0.05; **: p < 0.01; ***: p < 0.001.

## Data Availability

The original contributions presented in the study are included in the article/**Supplementary Material**. Further inquiries can be directed to the corresponding author.
